# Fortified Withaferin A accelerates the transition from fibrovascular to bone remodeling phase during endochondral bone formation to promote ossification

**DOI:** 10.3389/fendo.2025.1540237

**Published:** 2025-04-25

**Authors:** Kunal Chutani, Nikhil Rai, Anirban Sardar, Anupama Yadav, Divya Rai, Anuj Raj, Bhaskar Maji, Shikha Verma, Ashish Kumar Tripathi, Geeta Dhaniya, Lal Hingorani, Prabhat Ranjan Mishra, Ritu Trivedi

**Affiliations:** ^1^ Division of Endocrinology, CSIR-Central Drug Research Institute, Lucknow, UP, India; ^2^ Academy of Scientific and Innovative Research (AcSIR), Ghaziabad, India; ^3^ Division of Pharmaceutics and Pharmacokinetics, CSIR-Central Drug Research Institute, Lucknow, UP, India; ^4^ Jawaharlal Nehru University, New Delhi, India; ^5^ Pharmanza Herbals Pvt Ltd., Anand, Gujarat, India

**Keywords:** Withaferin A, parathyroid hormone, delayed union, menopause (estrogen withdrawal), bone mineralization, Young’s modulus

## Abstract

**Introduction:**

This study shows that Fortified Withaferin A (FWA, 10% w/w) accelerates bone healing, advancing from the fibrovascular to bone remodeling stage within 12 days, compared to the typical 23–24-day healing time in rodents. FWA (10% w/w) outperformed parathyroid hormone (PTH) in osteoclast regulation and minimized recovery time, highlighting its potential as a therapeutic agent for bone health.

**Methods:**

FWA (10% w/w) was administered orally at 125 mg·kg^–1^. A transverse osteotomy model was used to assess post-natal bone regeneration. Additionally, an estrogen-deficient model was employed to evaluate the therapeutic potential of FWA (10% w/w). Bone regeneration was validated through calcein incorporation, gene expression analyses, micro-CT imaging and mechanical testing. Pharmacokinetic profiling was used to determine plasma exposure and trough concentration.

**Results:**

FWA (10% w/w) effectively downregulated bone-resorbing genes, promoted anabolic responses, and reduced inflammation. It enhanced post-natal bone regeneration, likely via Runx-2 activation and modulation of osteogenic genes, alongside suppression of E3 ubiquitin-ligases Smurf1 and Smurf2, resulting in significantly enhanced callus formation and healing speed. Micro-CT revealed an enhanced callus area of ~95.14% within 12 days, compared to ~72.87% associated with normal healing. In the estrogen-deficient model, FWA (10% w/w) led to ~83.88% bone volume fraction at 23 days, exceeding the ~76.80% in controls and matching PTH effects. Material stiffness showed significant gains, with average Young’s modulus rising from ~54 ± 1.03 MPa to ~63 ± 2.54 MPa. Pharmacokinetic profiling indicated plasma exposure at 226 ng/ml*hr and higher trough concentration at 24 hr, contributing to optimum therapeutic effectiveness.

**Discussion:**

These results demonstrate that FWA (10% w/w) could significantly enhance bone mineralization and healing, facilitating an earlier transition from fibrovascular tissue to bone remodeling. The enhanced results, such as increased healing, better callus formation, and improved mechanical properties, indicate that FWA (10% w/w) is a potential intervention for delayed healing, especially in osteoporotic fractures.

## 1 Introduction

Fractures are traumatic injuries that many times require hospitalization. In this light, fragility fractures pose a problem as they are common in postmenopausal women (experiencing estrogen withdrawal) or women who have undergone hysterectomy with bilateral oophorectomy (1), resulting in weakened bones from reduced bone density. The drop in estrogen levels significantly accelerates bone loss, leading to a condition known as osteoporosis and, thus, susceptibility to frequent fractures (2). Without sufficient estrogen, bone resorption outpaces bone formation, resulting in thinner, more fragile bones. In postmenopausal women, fragility fractures frequently occur in the spine, hip, and wrist. Literature indicates that estrogen deficiency reduces callus formation and alters the healing process, resulting in a longer overall recovery time (3), as a balanced inflammatory response is important for successful fracture healing. These fractures contribute to long-term morbidity, disability, and even mortality in women.

A non-healing fracture is generally treated surgically to restore proper anatomical alignment and maintained with rigid internal fixation (4). Using rigid compression plates ensures tight alignment, promoting intramembranous healing, where bone regenerates without forming a callus by directly differentiating mesenchymal progenitors into osteoblasts. However, most fractures heal through endochondral ossification, which involves bridging the fracture gap with cartilage produced by chondrocytes before osteoblasts form bone. Bone repair is complete when the disorganized bone is remodeled into mature lamellar bone. Bone healing is a complex and dynamic process involving coordinating multiple cell types and communication networks. It involves three distinct phases: inflammation, repair, and remodeling (5, 6). The process begins with an accumulation of immune cells at the injury site, forming a fracture hematoma that initiates inflammation and promotes angiogenesis to recruit progenitor cells from the endosteum and periosteum (7). Postmenopausal osteoporosis has been considered a chronic inflammatory disease and thus increased levels of proinflammatory cytokines (8).

Progenitor cells like mesenchymal stromal cells (MSCs) and skeletal stem cells (SSCs) are essential in fracture healing. This primarily involves an attempt to reestablish continuity between the fracture fragments wherein osteoblasts derived from mesenchymal stromal cells (MSCs) form osteoid or callus on the exposed bone surface and establish a new haversian system through intracortical remodelling (9). Within the fracture site, callus formation occurs by endochondral ossification. Chondrocytes derived from MSCs in the periosteum and endosteum undergo hypertrophy, causing the cartilage matrix to become calcified (10, 11) later, to which they undergo apoptosis and osteoblasts take over to induce bone formation. Given these complexities, the various stages of fracture healing offer opportunities for therapeutic interventions, including preclinical evaluation of pharmacological agents and the investigation of the molecular mechanisms underlying the bone repair process. Therefore, identifying potential molecules aimed at enhancing the fracture healing process in osteoporotic conditions was the objective of our study, as in osteoporotic fractures, systemic inflammatory response during healing is generally higher compared to normal fractures, thus delaying the process or even non-union.

In our lab, we have previously demonstrated and established that Withaferin A (WFA), a steroidal lactone, exhibits potent anti-obesity properties (13). This is primarily achieved by binding and inhibiting the specific catalytic β subunit of the 20S proteasome. WFA also positively impacts bone formation by promoting osteoblast proliferation and differentiation, and in osteoclasts, it reduces cell numbers by downregulating the expression of tartrate-resistant acid phosphatase. This is achieved by virtue of its role in modulating the E3 ubiquitin ligase expression.

In our current investigation, we have elucidated the correlation between fracture healing and gene expression in normal and pathophysiological conditions of estrogen loss (menopause), establishing that the inflammatory phase and transition from fibrovascular tissue to bone remodelling are notably accelerated during fracture repair. Our findings reveal that Fortified Withaferin A extract (FWA, 10% w/w) significantly mitigates systemic inflammation and downregulates bone-resorbing genes, resulting in faster bone repair compared to the prolonged healing observed in menopausal fracture recovery. Additionally, Fortified Withaferin A extract (FWA, 10% w/w) addresses the challenges associated with the high cost of pure Withaferin A, making it a more viable option for positioning Withaferin A as a therapeutic agent.

## 2 Methods and materials

### 2.1 Animal and experimental study

All animal care and experimental procedures were conducted following approval from the Institute Animal Ethics Committee of the Central Drug Research Institute for the respective animal studies:

- SD rats (Animal ethics approval number: IAEC/2022/50/Renew-0/Dated-05/07/2022 (IAEC/2022/148/Renew-0/Dated-15/11/2022), and IAEC/2023/66/Renew-0/Dated-20/03/2023).

All the animals were housed in controlled conditions with a temperature of 24°C and 12-hour light-dark cycles. They were provided with ad libitum diet and water. At the conclusion of the study, all animals were individually weighed and sacrificed (12).

### 2.2 Osteotomy model

A total of 221 adult Sprague-Dawley rats were randomly assigned to three different studies.

Study 1: Dose-dependent effect of FWA enrichment on callus formation Eighty rats were divided into five groups (n=8-10 per group): Control (Fractured), PTH (20 µg, thrice a week; Positive Control), and FWA-enriched treatment groups (2.5%, 5%, and 10% at doses of 75, 125, and 250 mg/kg).

Study 2: Time-dependent effect of FWA (10% w/w) on early bone regeneration Sixty-nine rats were assigned to three groups (n=23 per group): Control, PTH, and FWA (10% w/w) at 125 mg.kg^-1^. The effect of FWA on fracture healing was assessed over a time course of 1-12 days.

Study 3: Effect of FWA (10% w/w) on delayed healing in an estrogen withdrawal model Seventy-two rats were used in this study (n=24 per group) and divided into three groups: Ovx (estrogen withdrawal model), Ovx + PTH, and Ovx + FWA (10% w/w) at 125 mg.kg^-1^. The study spanned 1-23 days to assess fracture healing in estrogen-deficient conditions.

After anaesthesia (Ketamine: Xylazine) in 3:1, the right hind leg was swabbed with 75% EtOH and a small skin 1 cm incision was made on the front skin of the mid-diaphysis of the femur to expose the targeted region for injury in SD rats. The surrounding muscles were cleared, and the periosteum was removed to expose the surface of the femoral bone. In order to create an injury, a drill hole was made in the anterior portion of the diaphysis, approximately 2 cm above the knee joint in SD Rats. The drill bit diameter used was 0.8 mm for rats. Upon recovery from anaesthesia, animals were housed individually with free access to food and water. Under these conditions, bone fractures heal through endochondral ossification at the centre of the fracture site, while intramembranous ossification occurs at the distal edges of the callus (13). The treatment was initiated on the following days and continued until the 12th day for the transverse osteotomy model and 23 days for the menopausal (estrogen withdrawal). At the end of the designated time period, the animals were sacrificed, and their femurs were collected to analyse bone microarchitectural parameters and dynamic histomorphometry study at the injury site (14).

### 2.3 Fracture site bone regeneration

Animals were given a single calcein dose of 20 mg.kg^-1^ of body weight through intraperitoneal administration 24 hours before they were sacrificed. The bones were embedded in acrylic material, and 50 µm sections were cut using an ISOMET bone cutter. Images were captured under a confocal microscope with appropriate filters for enhanced visualization to analyse the intensity of calcein binding to the fracture site, indicating new mineral deposition using Image J software.

For quantifying the microarchitecture of the callus formed in the drill hole, we performed µCT analysis using a Sky Scan 1276 CT scanner (Aartselaar, Belgium). The soft tissues were meticulously cleared without disturbing the bone callus and scanned using a 70kV, 200mA X-ray source and 1mm aluminium filter with a pixel size of 21.1 µm for rats for image acquisition. The images were reconstructed with the Sky Scan N recon and CTan software (1.7.4.6 and 1.18.8.0, Sky Scan, Bruker). For each fractured femur, the fracture line was identified using Dataviewer (SkyScan), and a volume of interest (VOI) was defined by selecting 100 axial slices centered on the fracture. The outer boundary of the callus—comprising bone, cartilage, and void space—was manually outlined in the 2D tomograms to determine the total callus volume (TCV). To assess maximum cortical bone density, 100 slices of the contralateral femur at the mid-diaphyseal region were analysed. The mineralized callus volume (MCV) was segmented using a density threshold of ~35% to ~57% of the maximum cortical density. Tissues below ~35% were considered non-mineralized. The total mineralized tissue volume, normalized to callus volume (TMV/TCV, %), used a range of ~35% to ~100% of the maximum cortical density, while bone volume normalized to callus volume (BV/TCV, %) was quantified with a range of ~57% to ~100% (13, 15).

### 2.4 Pharmacokinetic study

In this study, a validated UHPLC-MS/MS method was used to analyse the pharmacokinetics of FWA (10%w/w), a fortified extract enriched with Withaferin A, while also containing minor amounts of Withanolide A, Withanolide B, and Withanone. The analysis was conducted following the oral administration of FWA (10%w/w) in Sprague Dawley rats (SD rats; n=6) at the dose of 75, 125 and 250 mg.kg^-1^. The 125 mg.kg^-1^ dose was identified as the effective therapeutic concentration based on its optimal fracture-healing response. Initially, the UHPLC-MS/MS method was validated with respect to specificity, linearity, precision, accuracy, and robustness to ensure reliable quantification of Withaferin A and other withanolides in plasma samples. The accuracy and precision of the constituents in rat plasma samples and other validated parameters have been represented in **Tables 1**, **2**. The inter and intraday precision and robustness are represented in **Tables 3**, **4**, respectively. For pharmacokinetic assessment, plasma samples were analysed using PKSolver 2.0 software through non-compartmental analysis following extravascular input. The linear trapezoidal method was applied to calculate pharmacokinetic parameters such as Cmax, Tmax, AUC, and t1/2, to ensure that drug absorption, distribution, and elimination are thoroughly evaluated. The concentration of each Withanolide was measured in ng/ml units, allowing direct comparison of their pharmacokinetic behaviour and confirming that FWA (10% w/w) is enriched with Withaferin A as its primary bioactive component, with minor contributions from other withanolides (16).

**Table 1:** The linear equation, linear range, and LLOQ of the Withaferin A, Withanolide A, Withanolide B and withanone.

Withanolides	Linear equation	Range (ng/ml)	r2	LOD (ng/ml)	LOQ (ng/ml)
Withaferin-A	y =32943x-2845.1	0.49-250	0.9992	0.12	0.25
Withanolide-A	y = 7236.3x + 3114.2	0.49-250	0.9993	0.49	0.98
Withanolide-B	y =32950X-2662.2	0.49-250	0.9992	0.49	0.98
Withanone	Y=7800.9x + 104298	3.90-1000	0.9997	–	–

*r^2^-correlation coefficient; LOD, Limit of Detection; LOQ, Limit of Quantitation; LLOQ, lower, limit of quantification.

**Table 2:** Accuracy and Precision of the constituents in rat plasma samples, (n = 6).

Analyte	Nominal concentration (ng/mL)	Average Area Raito (± SD)	Extraction recovery (% ± SD)	Precision (RSD, %)	Accuracy (RE, %)
Withaferin A	1.95	0.20±0.01	95.79	2.61	1.87
	15.63	1.20±0.02	100.61	1.61	15.73
	125.00	9.11±0.22	100.01	2.39	125.02
Withanolide A	1.95	0.20±0.02	90.44	7.61	1.76
	15.63	0.93±0.03	101.25	3.46	15.82
	125.00	6.62±0.27	99.91	4.06	124.88
Withanolide B	1.95	0.12±0.00	105.60	2.98	2.06
	15.63	1.12±0.02	98.73	2.06	15.43
	125.00	9.29±0.60	100.02	6.46	125.02
Withanone	1.95	0.18±0.01	98.60	2.78	1.92
	15.63	0.98±0.04	99.83	1.87	15.60
	125.00	9.38±0.38	100.51	2.37	125.63

**Table 3:** Intra and inter-day precision and accuracies of the analyte in rat plasma samples, (ng/mL).

Analyte	Concentration (ng/ml)	Intra-day 01 (n = 6)		Intra-day 02 (n = 6)		Intra-day 03 (n = 6)		Inter-day (n = 6x3)	
		Average Area Ratio	% RSD	Average Area Ratio	% RSD	Average Area Ratio	% RSD	Average Area Ratio	%RSD
Withaferin A	0.98	0.13	3.11	0.13	4.71	0.13	1.54	0.13	4.71
	1.95	0.20	2.53	0.20	2.57	0.18	7.61	0.20	2.57
	15.63	1.18	4.15	1.19	1.83	1.15	2.62	1.19	1.83
	125.00	9.38	2.60	9.02	2.82	8.86	2.39	9.02	2.82
Withanolide A	0.98	0.09	8.69	0.09	9.54	0.10	8.93	0.09	9.54
	1.95	0.17	10.12	0.18	10.55	0.17	9.47	0.18	10.55
	15.63	0.88	3.20	0.94	2.93	0.79	4.98	0.93	2.93
	125.00	6.62	2.45	6.59	4.65	5.68	2.67	6.59	4.65
Withanolide B	0.98	0.86	4.24	0.91	2.58	0.87	1.78	0.91	2.58
	1.95	0.93	3.41	0.99	3.44	0.87	3.17	0.99	3.44
	15.63	1.98	4.92	2.04	3.73	1.83	1.96	2.04	3.73
	125.00	10.47	4.35	10.43	4.38	9.45	1.91	10.43	4.38
Withanone	0.98	0.78	4.12	0.75	2.46	0.72	1.62	0.89	2.36
	1.95	0.91	2.98	0.87	3.32	0.68	3.12	0.83	3.31
	15.63	1.85	4.57	1.98	3.68	1.73	1.93	2.12	3.62
	125.00	9.92	4.28	10.26	4.27	9.56	1.89	10.29	4.18

**Table 4:** Robustness; column temperature.

Withaferin A								
Column oven: 35^0^C			Column oven: 40^0^C			Column oven: 45^0^C		
Average Area Ratio	SD	%RSD	Average Area Ratio	SD	% RSD	Average Area Ratio	SD	% RSD
1.11	0.03	2.26	1.15	0.02	1.53	1.19	0.02	1.53
Withanolide A								
Column oven: 35^0^C			Column oven: 40^0^C			Column oven: 45^0^C		
Average Area Ratio	SD	% RSD	Average Area Ratio	SD	% RSD	Average Area Ratio	SD	% RSD
0.64	0.03	4.35	0.76	0.02	3.19	0.73	0.02	2.81
Withanolide B								
Column oven: 35^0^C			Column oven: 40^0^C			Column oven: 45^0^C		
Average Area Ratio	SD	% RSD	Average Area Ratio	SD	% RSD	Average Area Ratio	SD	% RSD
1.84	0.06	3.25	1.73	0.07	4.05	1.66	0.05	2.89
Withanone								
Average Area Ratio	SD	% RSD	Average Area Ratio	SD	% RSD	Average Area Ratio	SD	% RSD
1.62	0.04	2.98	1.53	0.05	3.98	1.46	0.03	2.35

### 2.5 RNA extraction and real-time PCR

We took the entire fracture site on the femur diaphysis and snap-frozen it in liquid nitrogen for the gene expression analysis. Frozen samples were then powdered with prechilled mortar and pestle and homogenised in 1ml Trizol RNAiso PLUS (Takara) following the manufacturer’s instructions. We used 500-1000 ng of RNA to reverse transcribed into cDNA with the High-Capacity cDNA Reverse Transcription Kit (Catalogue- 4368814 Thermo Fisher). Following this, quantitative real-time PCR (qRT-PCR) was performed in triplicates using PowerUP SYBR Green Master Mix (Thermo Fisher) on a QuantStudio 3 system (Applied Biosystems, Thermo Fisher, Mississauga, ON, Canada), in accordance with the manufacturer’s protocol. Gene transcript levels (as listed in **Table 5**) were assessed using the comparative Ct (2-ΔΔCt) method (17, 18). Primers were designed using Primer-BLAST (National Centre for Biotechnology Information [NCBI], Bethesda, MD, USA) and are listed in **Table 5**.

**Table 5:** List of primers and their sequences used in the study.

Gene Symbol	Gene Name	Primer Sequence	Accession No.
β - actin	β-actin	F–CTCCCTGGAGAAGAGCTATGA R–AGGAAGGAAGGCTGGAAGA	NM_031144.3
BMP2	Bone morphogenetic protein2	F– CGGCTGCGGTCTCCTAA R–GGGAAGCAGCAACACTAGA	NM_017178.2
Sox9	SRY Box Transcription Factor 9	F–GTACCCGCATCTGCACAAC R–CTCCTCCACGAAGGGTCTCT	NM_080403.3
Col2a1	Collagen Type 2, α1 chain	F- GGATGTATGGAAGCCCTCGTC R-GTGACCCTTGACACCAGGAA	NM_001414896.1
Col1a1	Collagen Type 1, α1 chain	F- CAAGATGGTGGCCGTTACTAC R- GCTGCGGATGTTCTCAATCT	NM_053304.1
Col10a1	Collagen Type X, α1	F- ATGGCTTCACAAAGAGCGGA R- CCTACCCAAACGTGAGTCCC	NM_013140.1
Runx2	Runt related transcription factor 2	F- TGGCCTTCCTCTCTCAGTAA R- GTAAGTGAAGGTGGCTGGATAG	NM_001278483.2
Ocn	Osteocalcin	F- TGACTGCATTCTGCCTCTC R- CGGAGTCTATTCACCACCTTAC	NM_013414.1
Acp5 (Trap)	Tartrate resistant acid phosphatase 5	F– GGAACCACAGAGGCTTACAT R– CCACTCCCAAGAAAGGTCTAC	NM_001270889.1
Opn (Spp1)	Osteopontin	F– CAGCCAAGGACCAACTACAA R–TGCCAAACTCAGCCACTT	NM_012881.2
Rankl	TNF Superfamily member 11	F–ACTGTCTGGACCTCGGTGAA R–CTGCGCTCGAAAGTACAGGA	NM_057149.2
CtsK	Cathepsin K	F- TCCTCAACAGTGCAAGCGAA R- CCAGCGTCTATCAGCACAGA	NM_031560.2
Acan	Aggrecan	F- AGCCCTTGTCTGAATGGAGC R- GTTGGTTTGGACGCCACTTC	NM_022190.2
Smurf 1	SMAD specific E3 ubiquitin protein ligase 1	F - CTGAAACCCAATGGCAGAAATG R – GGGCTTCGATTCCTCTCATAAA	NM_001109598.1
Smurf 2	SMAD specific E3 ubiquitin protein ligase 2	F - CCAGACTAGCAGAGAGAAGAGT R - TAGGTCTGGAGGAGTGTGTAAG	XM_039086171.2

### 2.6 Histology and histomorphometric analysis

Bone samples were decalcified in 10% EDTA (Sigma-Aldrich) for three weeks, and the solution was changed every three days. Decalcified mice bone samples were further embedded in paraffin wax before the staining procedure. Sections were then sectioned to 7-micron thickness and stained with Safranin O/Fast green to determine cartilage and bone. The proportions of cartilage and bone were quantitated by determining the areas that stained positive for Safranin O (red, indicating proteoglycan content) and Fast Green (blue, indicating collagen), respectively. This was done through thresholding and then manually fine-tuning the region of interest, with results expressed as a percentage of the entire callus area. Tartrate-resistant acidic phosphatase (TRAP) staining was performed by immersing them in a pre-warmed TRAP staining solution mix for 30 minutes. After rinsing with distilled water, a 0.02% fast green counterstain was applied. The tissue sections were dehydrated using graded alcohols and cleared in Xylene before mounting for observation under a light microscope. Osteoclasts were identified as TRAP-positive cells with at least three nuclei (19, 20).

### 2.7 Mechanical testing of bone

The micro-indentation tests were performed at the fracture site in the femur diaphysis using the MACH-1 TM v500css micromechanical system (Bio momentum, Laval, Quebec, Canada). The tests were conducted under displacement control using a spherical 1mm diameter ruby tip. Force–displacement data were measured with a 1.5 N load cell and the linear stage’s optical encoder (0.1 µm resolution) at a sampling rate of 200 Hz. To position the bone for indentation, the indenter was mounted using a custom fabricated adaptor attached to an XY positioning stage (Newport motion controller ESP302, linear XY stage, Newport Corporation, Irvine, California) with a micrometre (1 µm precision). The Basler camera (1.3MPX, MA732) was utilized to identify the region of interest for micro-indentation testing. All indentations were conducted at room temperature, and each sample was rehydrated with phosphate-buffered saline prior to testing (21, 22). Tissue mechanical properties, such as Young’s modulus (E), Bulk modulus (K) and toughness (I), were calculated.

### 2.8 Statistical analysis

Data are presented as means ± standard error of the mean (SEM) unless otherwise specified, with sample sizes detailed in the figure legends. To assess genotype differences across time points in micro-CT, gene expression analysis, osteoclast number, mechanical testing data, mineralized callus volume by total callus volume, a one-way ANOVA followed by Tukey’s multiple comparisons test was conducted using GraphPad Prism 8.4.2 (GraphPad, La Jolla, CA, USA). For qualitative analysis of immunostaining data, two fields of view (FOVs) per animal and three animals per genotype were included, with representative results presented. Statistical significance was set at (***p< 0.001, **p 0.002, *p 0.033).

## 3 Results

### 3.1 Effect of different doses and strength of FWA (10% w/w) at the fracture site in healthy rats

**Figure 1:**
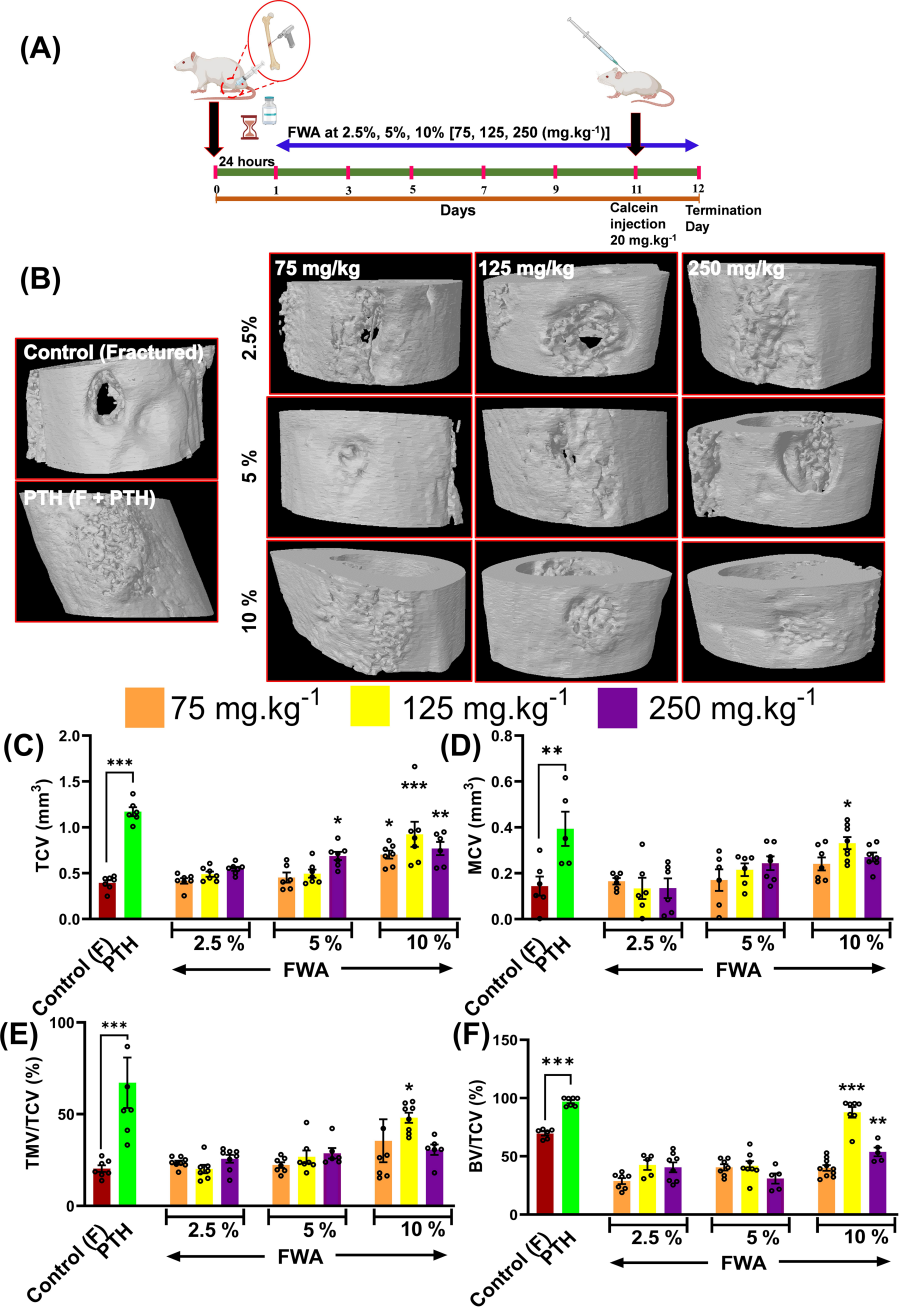
Effects of Withania somnifera enriched extract (FWA) on bone healing in a transverse osteotomy model. **(A)** In a drill hole injury model (transverse osteotomy), The experimental design depicts the timeline for administering FWA at varying concentrations (2.5%, 5%, and 10%, corresponding to 75, 125, and 250 mg.kg^-1^). Calcein injection was carried out on day 11 to label mineralizing bone, and the SD rats were sacrificed on day 12 for analysis. **(B)** Micro-CT images of fractured femurs from different treatment groups, including controls (both untreated and treated with parathyroid hormone [PTH]) and those treated with various concentrations of FWA. The images display the progression of bone healing and callus formation at the fracture site. **(C-F)** Quantitative analysis of tissue callus volume (TCV), mineralized callus volume (MCV), total mineralized volume normalized to callus volume (TMV/TCV), and bone volume/total callus volume (BV/TCV) in the fractured femurs across different groups. The data are presented as mean ± SE (n = 6). Significant differences compared to control groups are indicated (***p< 0.001, **p 0.002, *p 0.033).

Osteotomies were produced, generating gaps of approximately 0.8mm that were not significantly different between the Control and Treatment groups when measured by Micro-CT and the experimental design of this schematic administration of FWA (10% w/w) at different concentrations is shown in (**Figure 1**). Fracture calluses were examined at different doses and at different enrichment strength (2.5, 5.0, and 10.0%) in the groups for 12 days by micro-CT post-fracture to determine the effects FWA (10% w/w) on changes in callus formation and mineralization. Comparisons were made with standard of care parathyroid hormone (PTH) that is known to regulate endochondral bone repair (**Figure 1**). Estimation of the total callus volume (**Figure 1**), mineralized callus volume, total mineralized tissue volume normalized to callus volume, and total normalized bone volume show that these indices were significantly increased in 125 mg.kg^-1^ dose as compared to the other doses. Peak mineralized callus volume was observed on day 12 of treatment with FWA (10% w/w) at 125 mg.kg^-1^ (10% w/w) by which time ossification of the callus is observed at the fracture site (**Figure 1**). When this was normalized to tissue callus volume the dose of 125 mg.kg^-1^ FWA (10% w/w) showed an increase in percentage of total mineralized callus tissue at compared to that of control in normal healing process (**Figure 1**). Data shows that the administration of FWA (10% w/w) at 125 mg.kg^-1^ and 250 mg.kg^-1^ resulted in the best response to callus formation that was comparable to PTH at the fracture site and evidence of new bone formation. Quantitatively, a significantly increased trabecular bone volume/tissue callus volume (BV/TCV) to ~87.76% (125 mg.kg^-1^) and ~53.78% (250 mg.kg^-1^) compared to the negative (control) ~30.09% and positive control (PTH) ~96.98% (**Figure 1**).

**Figure 2:**
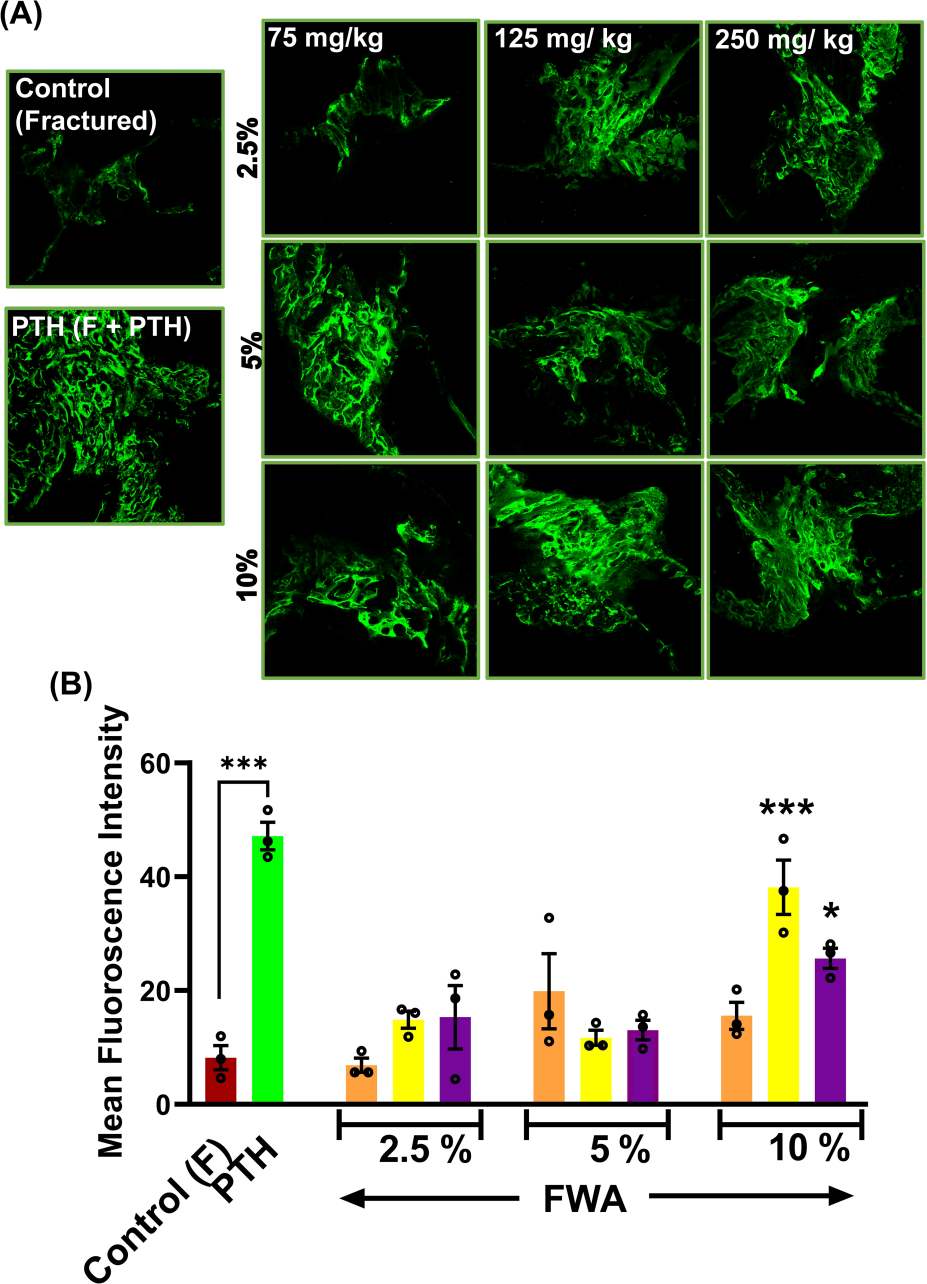
Effects of Withania somnifera enriched extract (FWA) on fracture healing and bone formation. **(A)** Fluorescence microscopy images of calcein-labeled sections from different treatment groups, illustrating new bone formation at the fracture site. Control (fractured), PTH-treated, and various doses of FWA-treated groups (2.5%, 5%, 10%) are shown for comparison. **(B)** Quantitative analysis of calcein fluorescence intensity, representing new bone formation in the different treatment groups. The data are presented as mean ± SE (n = 6). Significant differences compared to control groups are indicated (***p < 0.001, *p 0.033). This figure demonstrates the positive impact of FWA on bone formation and fracture healing, as evidenced by improved structural parameters and increased new bone formation in treated groups compared to fractured control animals.

The calcein labelling intensity observed in newly formed callus at the drill hole site during fracture healing reveals that the most robust bone-regeneration occurred with FWA (10% w/w) at a dosage of 125 mg.kg^-1^, which was found comparable to the positive control (PTH 20 µg/3 days a week alternatively) (**Figures 2A, B**).

The micro-CT and the calcein labelling intensity at the callus site suggest that administering FWA (10% w/w) at 125 mg.kg^-1^ was the most effective dose for repairing the fracture as early as day 12. This data suggests that FWA could reduce the therapeutic window to half (day 12) compared to the normal healing process of 21-35 days. This dose and timepoint were taken up for further experiments.

### 3.2 Pharmacokinetic profiling of optimized FWA formulation in SD rats

**Figure 3:**
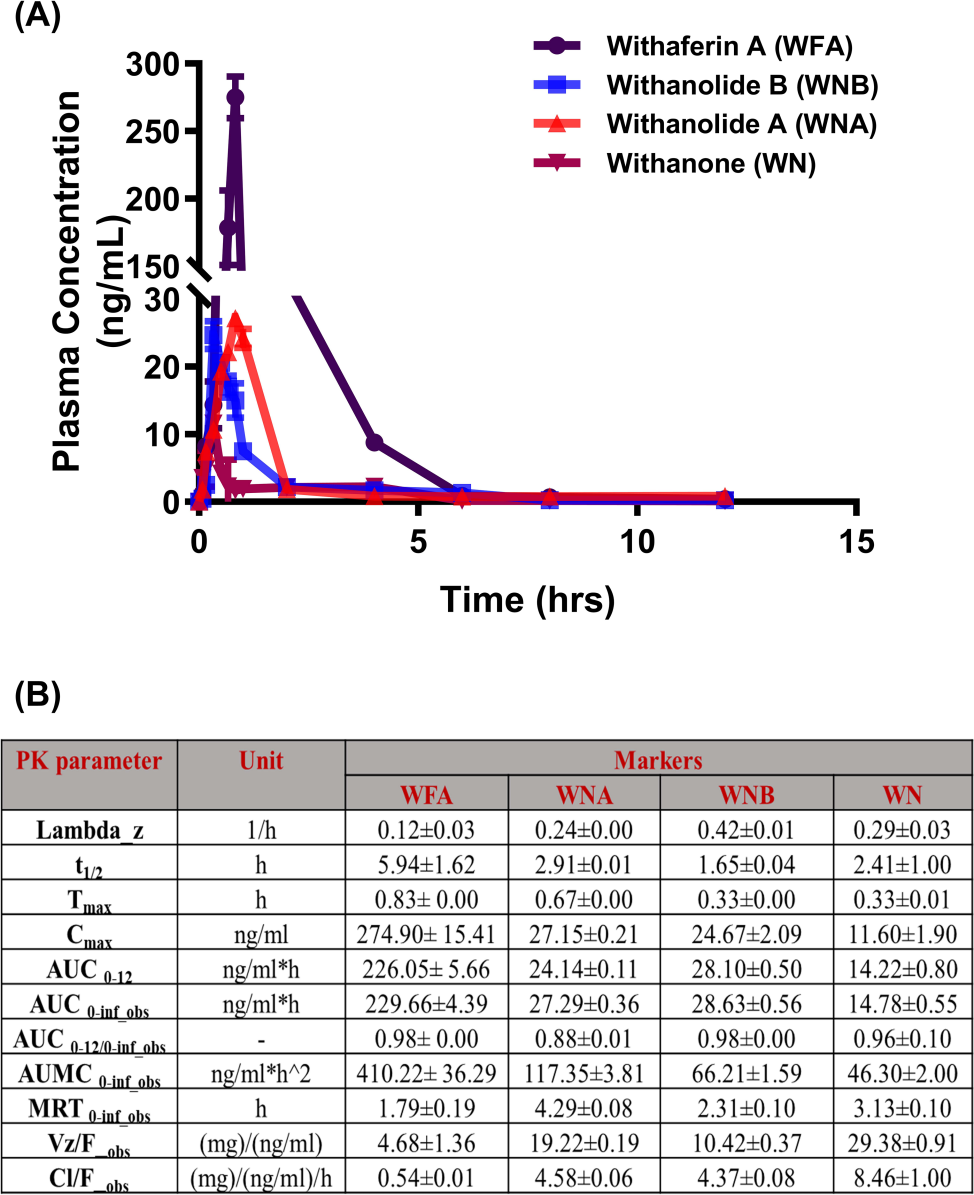
Pharmacokinetic profile of Withaferin A and related withanolides in SD rats. **(A)** Mean plasma concentration-time plots of four withanolides (WFA, WNA, WNB, and WN) after oral administration of Withania somnifera extract (FWA) at the dose of 125 mg.kg^-1^ (10% w/w) in Sprague Dawley rats (Mean ± SD, n = 6). are represented in different colours: WFA (purple), WNA (red), WNB (blue), and WN (pink). The data points reflect mean concentrations ± SE (n = 6). **(B)** The table presents the pharmacokinetic (PK) parameters of WFA and related withanolides. The parameters are presented with their units and values for each Withanolide (WFA, WNA, WNB, WN) showing mean ± SE (n = 6). This figure offers a comprehensive overview of the pharmacokinetic behaviour of Withaferin A and its related compounds in SD rats, highlighting their absorption, distribution, metabolism, and elimination characteristics in the rat model.

Having proven the efficacy dose of FWA (10% w/w) to be 125 mg.kg^-1^, we wanted to establish the pharmacokinetic profile of the FWA (10% w/w) formulation. The C*_max_* of Withaferin A (WFA) was found to be 274.90± 15.41 ng/ml, which is substantially higher as compared to other markers like Withanolide A, Withanolide B, and Withanone. Similarly, the AUC_0-12_ of Withaferin A was found to be 226.05± 5.66 ng/ml*h, which is nearly nine-fold higher as compared to other withanolides (**Figure 3**), suggesting that the FWA (10% w/w) formulation was indeed enriched with WFA as its primary bioactive component. Additionally, we assess the other withanolides present in FWA (10% w/w) that might have imparted therapeutic efficacy after *in vivo* administration. Data shows that Withanolide A, Withanolide B, and Withanone were rapidly absorbed from the gastrointestinal tract. The concentration of different withanolides; Withaferin A, Withanolide A, Withanolide B, and Withanone in the bloodstream over 12 hours following a single dose of 125 mg.kg^-^1 is represented in the plasma concentration-time curve overlay graph (**Figure 3**). The y-axis is on a logarithmic scale, ranging from 0.1 to 300 ng/mL, and the x-axis represents time in hours. At 0 hours, the concentration of Withaferin A starts low, marking the baseline. Within the first hour, a sharp increase, peaking slightly above 100 ng/mL, indicating rapid absorption. After this peak at around 1 hour, the concentration declines noticeably, reaching about 10 ng/mL by the 4-hour mark. The decline continues slowly from 4 to 12 hours, with the concentration approaching 1 ng/mL by 12 hours, reflecting a slower elimination phase. This data conforms to the fact that Withaferin A is responsible for the improved efficacy in the rapid fracture model. These results suggest that the time required to reach maximum concentration (T*_max_*) and plasma half-life (t_1/2_) of Withaferin A was found to be 0.83 h and 5.94 ± 1.62 h, respectively, while for Withanolide A and B, t_1/2_ were found to be 2.91 ± 0.01 h and 1.65 ± 0.04 h respectively. The data also agrees with the fact that molecules like Withaferin A having reported log P value (3.86) less than 5 and Total polar surface area (TPSA) of ~96 are well absorbed, thus showing the improved activity. These pharmacokinetic properties establish FWA (10% w/w) as a Withaferin-A enriched extract with optimized bioavailability.

### 3.3 Longitudinal assessment of callus formation in healthy SD rats using the effective dose of FWA (10% w/w), compared to the standard of care PTH

**Figure 4:**
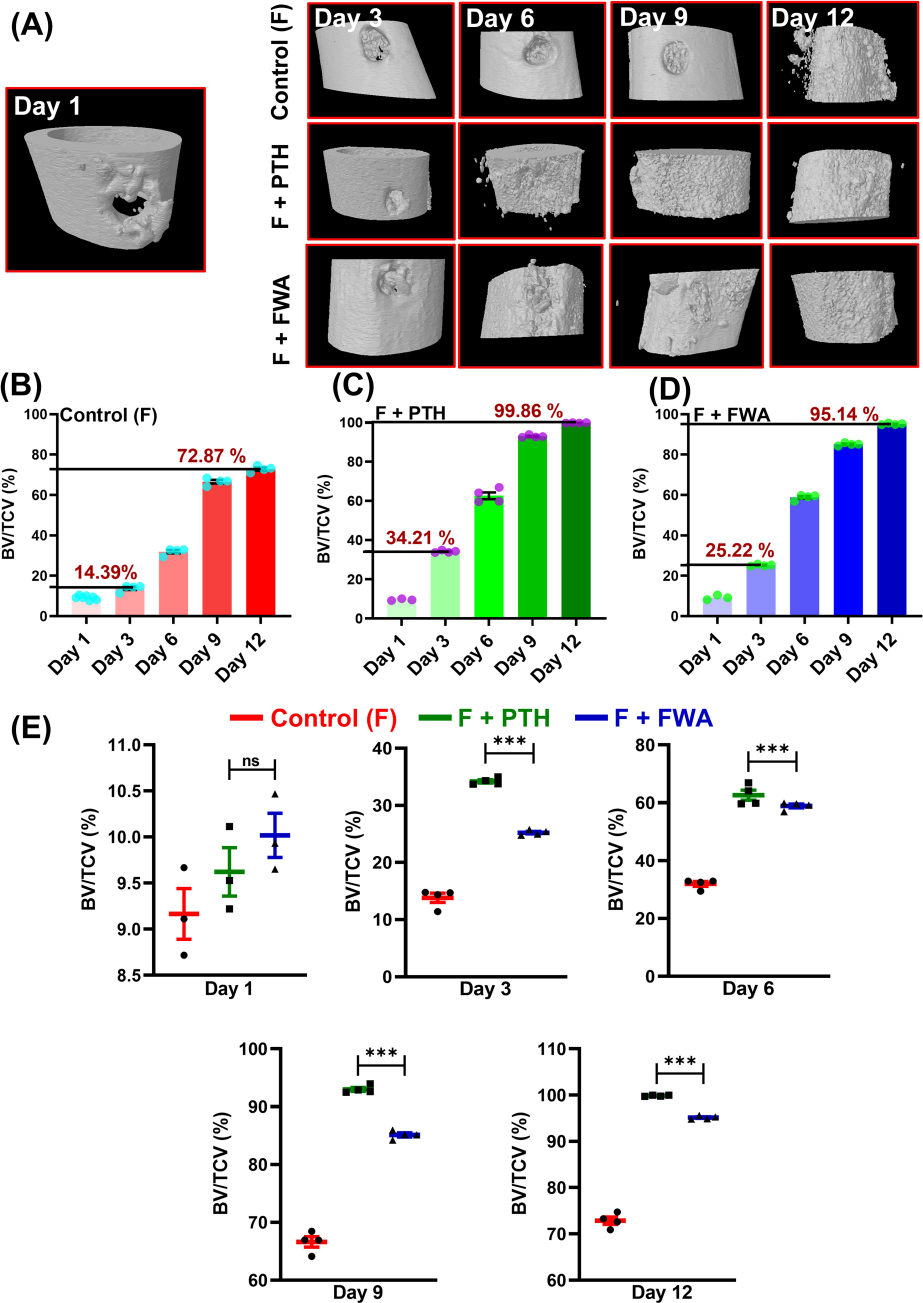
FWA promotes fracture healing in the transverse osteotomy model (Drill hole injury) in time specific manner. **(A)** 3-D representation of the bone microarchitecture as obtained from µCT analysis. **(B)** Quantitative measures of the parameter bone volume/tissue callus volume (BV/TCV, %) between control (fractured) group **(C)** PTH treatment in fractured SD rats with comparison to **(D)** FWA treatment **(E)** Values of BV/TCV, % with respect to healing are represented in time specific manner. Data are represented as mean ± SEM compared to the control group (***p < 0.001). ns, non-significant (ns p = 0.47).

The impact of FWA (10% w/w) at the effective dose of 125 mg.kg^-1^ was examined time-dependently over 12 days by micro-CT. This helped us determine the effects of increased levels of FWA (10% w/w) in a longitudinal fashion in callus formation and mineralization. FWA (10% w/w) at 125 mg.kg^-1^ showcases a progressive enhancement in BV/TCV (%) in the duration of healing, culminating in complete restoration by day 12. A 3D µ-CT visualization elucidates the complex process of *in-vivo* bone regeneration, highlighting the efficacy of FWA (10% w/w) at 125 mg.kg^-1^ from day 1 to 12. (**Figure 4**). The BV/TCV significantly increased by day 6 in the control (Fractured group); however, in the FWA (10% w/w) and the PTH treatment groups, BV/TCV increased as early as day 3, reaching its peak by day 12. Femoral data analysis reveals that in the control fractured group, BV/TCV (%) increased from approximately (14.39%) on day 3 to a notable (~72.87%) by the end of day 12 (**Figure 4**), aligning with typical fracture healing timelines. In contrast, administering PTH and FWA (10% w/w) at 125 mg.kg^-1^ demonstrated remarkable enhancements in BV/TCV. On day 3, PTH exhibited a striking increase of approximately (34.0%) (**Figure 4**), while FWA (10% w/w) showed a substantial rise of approximately (25.0%) (**Figure 4**). By day 12, these interventions maintained their efficacy, with PTH and FWA (10% w/w) showing further increases of approximately (99.86%) and (95.0%) respectively. **Figure 4** illustrates bone volume to tissue callus volume (BV/TCV %) across different time points for three groups—Control (F), PTH treatment, and FWA (10% w/w) treatment. These findings underscore the potential of PTH and FWA (10% w/w) as accelerators of fracture healing, surpassing the typical healing trajectory (23). In summary, the results suggest that, like PTH treatment, FWA (10% w/w) is equally effective in promoting bone- regeneration and improving bone quality. The control group with no additional treatment exhibited minimal bone repair.

### 3.4 Time-dependent gene expression analysis of healing at the fracture site

**Figure 5:**
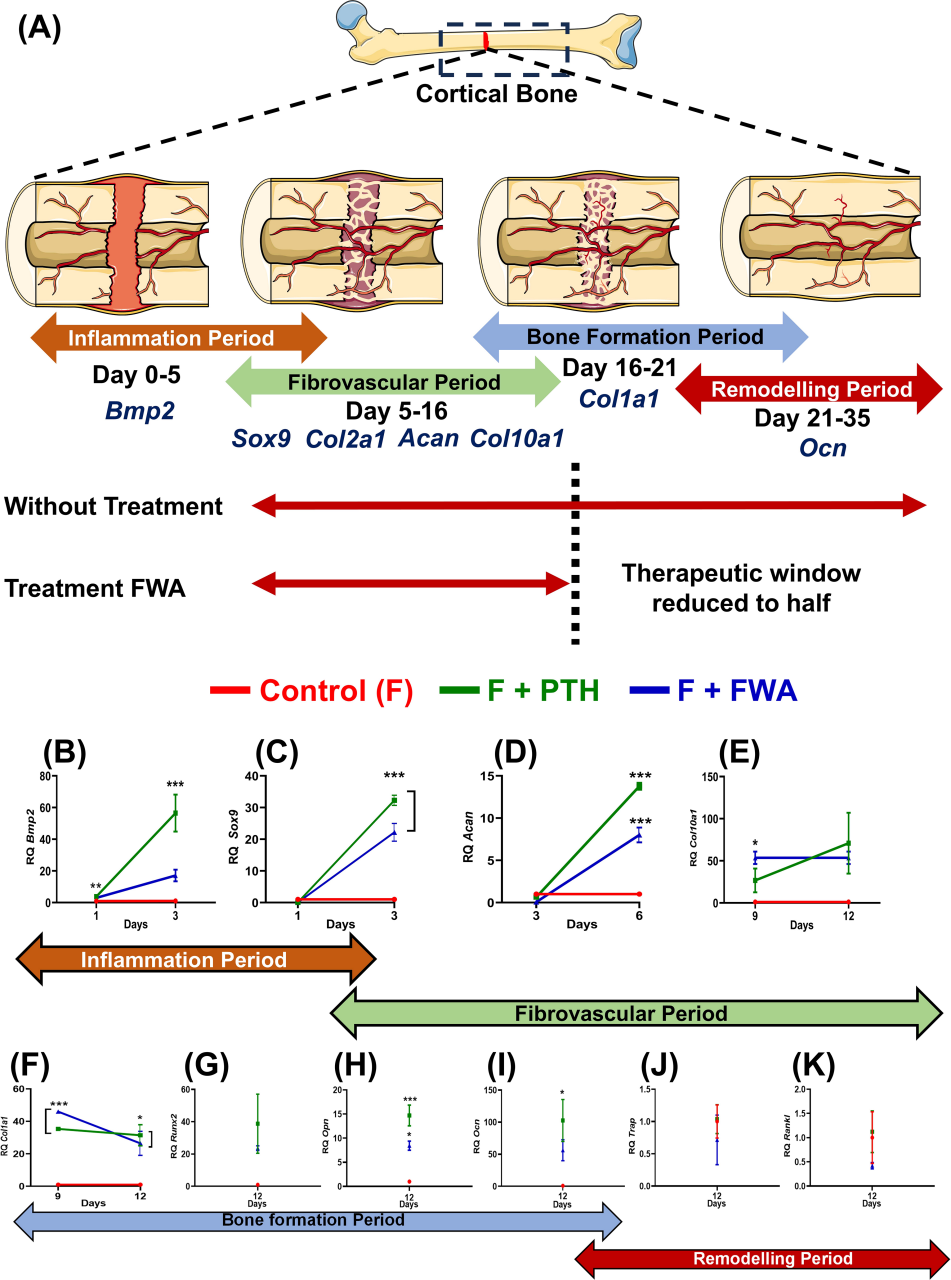
Phases of bone healing post drill hole injury in the femur diaphysis. **(A)** Schematic representation of the four phases of bone healing: Inflammation phase (Days 1-6), Fibrovascular phase (Days 3-12), Bone formation phase (Days 9-18), and Bone remodeling phase (Day 18-23). Key regulatory markers for each phase are indicated: BMP2 for inflammation, Sox9, Col2a1, ACAN, and Col1a1 for the fibrovascular period, Runx2, Col10a1, Ocn, and Opn for bone formation, and RANKL, Cathepsin K, and TRAP for remodeling. Quantitative analysis of gene expression levels over time for different treatment groups: Control (F, red), F + PTH (green), and F + FWA (blue) is shown with respect to different phases BMP2 **(B)**, Sox 9 **(C)**, Acan **(D)**, Col10a1 **(E)**, Col1a1 **(F)**, Runx2 **(G)**, Opn **(H)**, Ocn **(I)**, Trap **(J)** and Rankl **(K)**. Data are represented as mean ± SEM compared to the control group (***p< 0.001, **p 0.002, *p 0.033).

Time-dependent changes in callus formation during fracture healing involve specific gene expression at each time point like, from Inflammation (Bmp-2) to Fibrovascular (Sox 9), (Col2a1, Acan, Col10a1 to the bone formation (Col1a1) to finally the remodeling period (OCN) when the bone is completely mineralized and repaired (**Figure 5**), our assessment at these timepoints shows that when the treatment groups were normalized with the control (F) group the Fracture (F) + PTH and F+FWA (10% w/w) group exhibited a time-specific increase, in the distinct phases of fracture repair.

Early on, periosteal cells responded to Bone Morphogenetic Protein 2 (BMP2) (**Figure 5**), leading to a significant ~57.0-fold increase in the PTH administered group and ~17.0-fold increase in the FWA (10% w/w) group, fostering chondrogenesis followed by osteogenesis. By *day 6*, angiogenic cells facilitated the formation of small blood vessels and fibrovascular tissue, with remarkable upregulation of genes observed in PTH and FWA (10% w/w) at 125 mg.kg^-1^. This was evident through substantial elevations in Sox9 (**Figure 5**) (~32.29-fold and ~22.16-fold, respectively), Aggrecan (Acan) (**Figure 5**) (~14.0-fold and ~8.0-fold, respectively), Col10a1 (**Figure 5**) (~71-fold and ~54.0-fold, respectively).

As the process progressed, markers indicative of endochondral bone formation and remodeled bone were expressed by *day 9* and *day 12* in PTH and FWA (10% w/w) at 125 mg.kg^-1^. Notable increases were observed in Col1a1 (**Figure 5**) (~32.0-fold and ~26.00-fold, respectively) in Runx2 (**Figure 5**) (~102.0-fold and ~56.0-fold, respectively), as well as mineralization markers like Osteopontin (Opn) (**Figure 5**) (~15.0-fold and ~8.0-fold, respectively) and Osteocalcin (Ocn) (**Figure 5**) (~38.0-fold and ~23.0-fold, respectively.

The expression analysis data of the remodeling phase when the bone is completely formed showed that the PTH did not affect the osteoclast activity. As we observed, no significant changes in the expression of the TRAP osteoclast-specific marker gene (**Figure 5**) (~1.0-fold), Rankl (**Figure 5**) (~1.0-fold, respectively). However, FWA (10% w/w) did affect the remodeling phase as we observed downregulation of osteoclast marker genes by approximately 50%, indicating a shift towards a less resorptive state, suggesting a trend towards a very normal healing process involving all the three cell types of chondrocytes forming callus and then the bone-forming cells osteoblast and bone-resorbing cells osteoclast.

### 3.5 Assessment of FWA (10% w/w) in estrogen withdrawal (menopause) model

**Figure 6:**
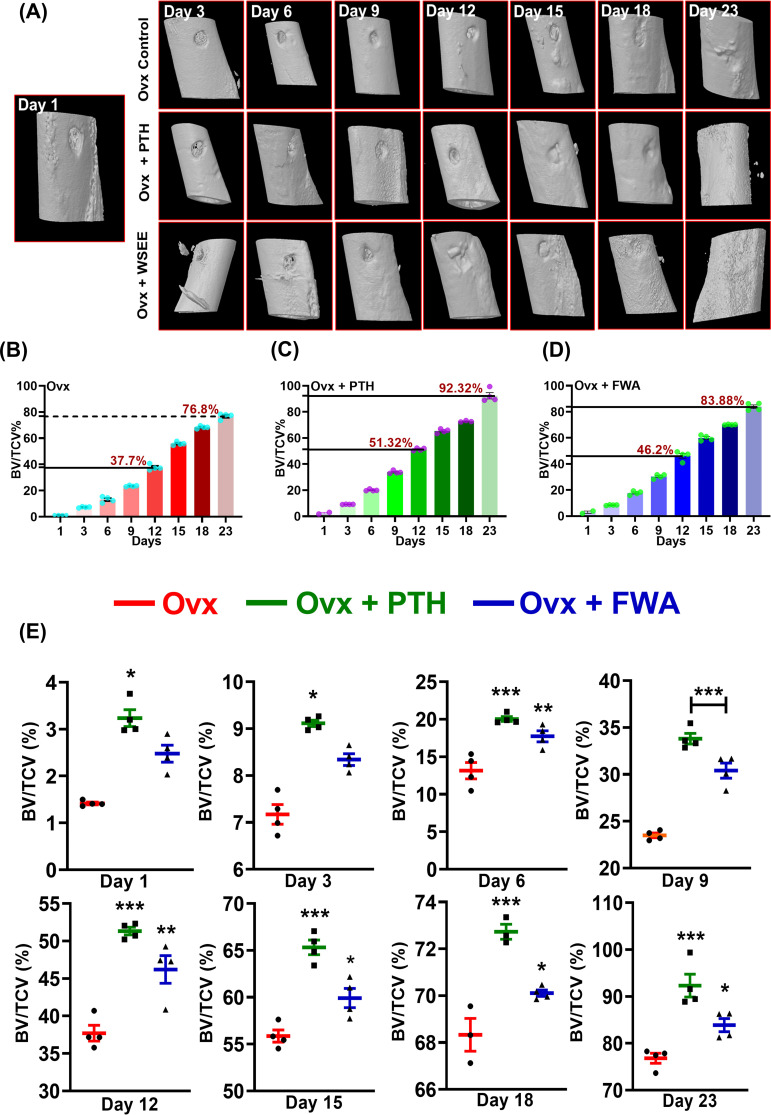
Quantitative assessment of bone regeneration in ovariectomized (Ovx) rats treated with PTH or FWA, focusing on bone volume to total callus volume (BV/TCV) measurements over time. **(A)** 3D reconstructed images from µ-CT scans showing the progression of bone healing in Ovx rats across different days (Day 3, 6, 9, and 12) for the Control (Ovx), Ovx + PTH, and Ovx + FWA treatment groups. The images illustrate the newly mineralized tissue formed at the fracture site over time. FWA treatment leads to faster and more extensive mineralization than PTH and control groups. **(B)** Quantification of µ-CT data of trabecular parameter represented in percentage of Bone volume/Tissue callus volume (% BV/TCV) in Ovx rats in comparison to different treatment with **(C)** PTH and **(D)** FWA. **(E)** Values of BV/TCV, % with respect to healing are represented in a time-specific manner. Data are represented as mean ± SEM compared to the control group (***p< 0.001, **p 0.002, *p 0.033).

As estrogen deficiency reduces callus formation and alters the healing process, resulting in a longer overall recovery time, we wanted to assess the efficacy of FWA in this pathophysiological condition. The effect of FWA (10% w/w) shows that BV/TCV (%) increased over time at the fracture site, ultimately resulting in maximum healing by day 23, even in the context of estrogen withdrawal. The three-dimensional µ-CT visualization illustrates the *in-vivo* bone-regeneration process under the influence of FWA (10% w/w), spanning from days 1 to 23 (**Figure 6**). Notably, bone volume normalized to tissue callus volume fraction (BV/TCV) exhibited a gradual increase over time, peaking on day 23, showcasing the efficacy of this intervention in facilitating bone healing, particularly in scenarios of delayed fracture repair.

Quantitative analysis in the osteoporotic fracture group for BV/TCV (%) illustrates ~ 37.0% on day 12 due to a delay in the healing process, compared to the expected ~73.0% in the normal fracture group by the same time frame. By day 23, this rose to ~77.0% (**Figure 6**). Contrastingly, in groups administered with PTH and FWA (10% w/w), BV/TCV (%) on day 12 showcased significant improvements, with PTH exhibiting a ~36% increase (51%) and FWA (10% w/w) showing a remarkable ~23% increase (46.0%) (**Figures 6C**). On day 23, BV/TCV continued to demonstrate enhancements, with PTH and FWA (10% w/w) displaying increases of approximately ~20% (92%) and ~9% (83%), respectively, compared to the prolonged 35-50 days typically required for delayed osteoporotic fracture healing processes (24). The administration of FWA (10% w/w) in the menopause (estrogen withdrawal) model showed a steady increase in BV/TCV (%) over the observed timeframe (**Figure 6**).

### 3.6 Histological and histomorphometric assessment of callus formation in estrogen withdrawal conditions

**Figure 7:**
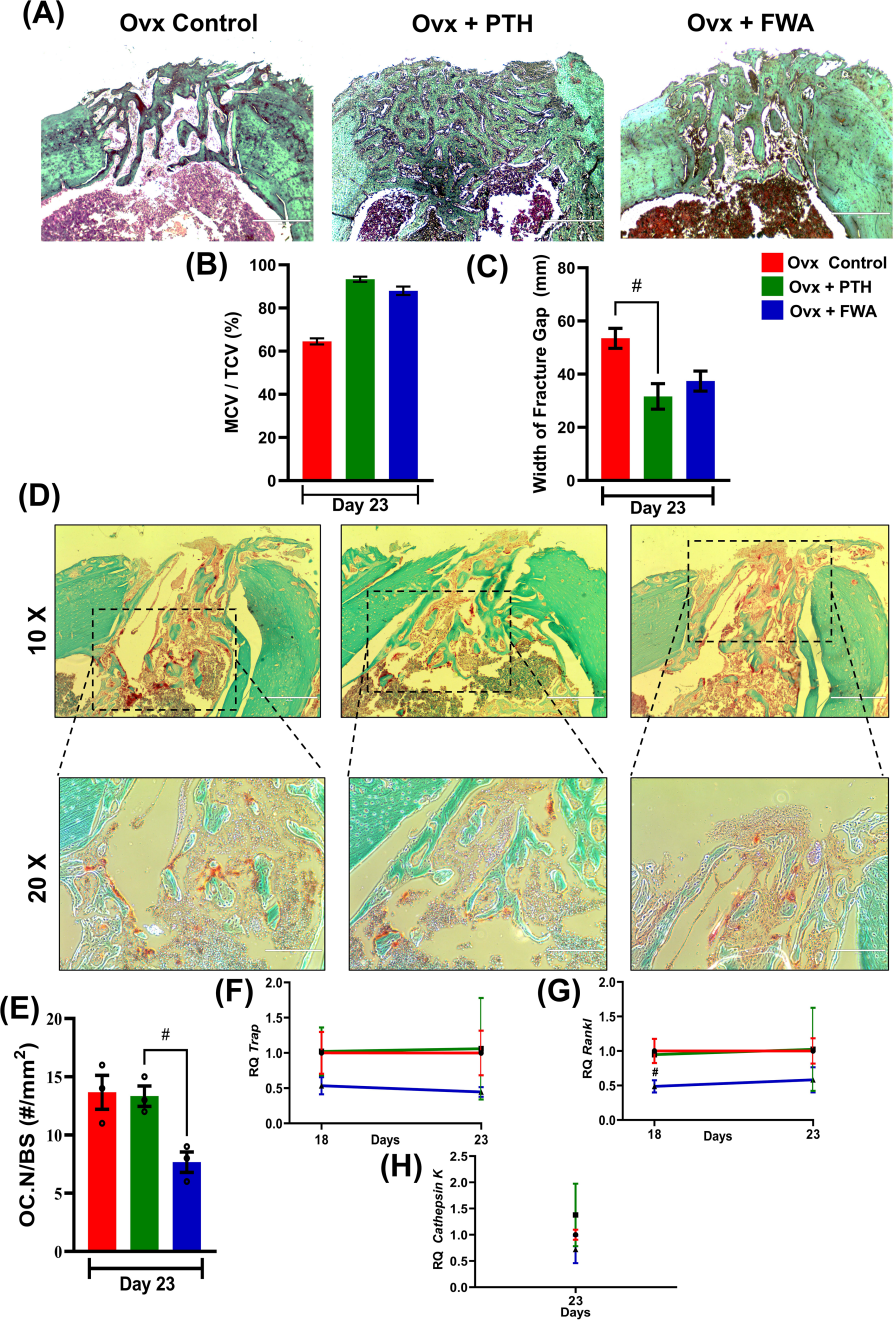
Histological and histomorphometric analysis of fracture healing in ovariectomized (Ovx) rats treated with PTH or FWA. **(A)** Representative images of fractured bone sections stained with Safranin O (to visualize cartilage) in Ovx Control, Ovx + PTH, and Ovx + FWA groups on Day 23. The images highlight differences in cartilage and bone remodeling among the groups. **(B)** Mineralized callus volume as a percentage of total callus volume (MCV/TCV): PTH and FWA treatments increased MCV/TCV compared to Ovx Control, indicating enhanced mineralization. **(C)** Quantification of the fracture gap width (mm) on Day 23. The Ovx control group exhibited the largest fracture gap, while both PTH and FWA treatments significantly reduced the fracture gap size. PTH treatment led to the smallest fracture gap, indicating superior bone regeneration. **(D)** Representative TRAP (Tartrate-resistant acid phosphatase) staining images of fracture callus sections on Day 23 from Ovx control (red), Ovx + PTH (green), and Ovx + FWA (blue) groups. The images at 10X and 20X magnification demonstrate osteoclast activity and bone resorption at the fracture site. **(E)** Quantification of osteoclast numbers per bone surface area (OC. N/BS, #/mm²) on Day 23. The Ovx control group showed the highest osteoclast numbers, while FWA treatments significantly reduced osteoclast activity compared to Ovx control and PTH groups. The FWA group exhibited the lowest osteoclast number, suggesting enhanced inhibition of bone resorption. Quantitative analysis of gene expression levels over time for different treatment groups: Control (F, red), F + PTH (green), and F + FWA (blue) is shown with respect to different phases for TRAP **(F)** RANKL **(G)**, Cathepsin K **(H)**. Data are presented as mean ± SEM, with statistical significance (^#^p 0.033) compared to the Ovx control group. # denotes statistically significant downregulation relative to the control.

To evaluate the proportions of cartilage and bone within the fracture callus, micro-CT analysis was complemented by histological assessment of Safranin-O/Fast Green–stained callus tissue sections (**Figure 7**). The percentage of mineralized callus volume (MCV) relative to the total callus volume (TCV) was highest in the Ovx + PTH group, followed by the Ovx + FWA (10% w/w) group, and was lowest in the Ovx control group. This indicates that treatment with FWA (10% w/w) and PTH promoted greater mineralization of the callus compared to untreated Ovx controls (**Figure 7**). FWA (10% w/w) and PTH treatment showed better outcomes in reducing fracture gap width, which was measured for bone- regeneration at the fracture site when administered with FWA (10% w/w) and PTH at day 23 (**Figure 7**). Quantitative histomorphometry revealed a ~36% increase in mineralized cartilage volume. Safranin O staining reveals differences in cartilage content between the groups. The control group Ovx revealed mostly red-stained regions, which indicated that a higher percentage of cartilage was contained in the fracture callus. In contrast, the group Ovx + PTH showed fewer Safranin O-positive regions, indicating a process progressing towards bone formation with a reduced cartilage content. The Ovx + PTH group had the least cartilage staining, indicating more advanced healing with a higher degree of endochondral ossification and matrix remodeling, and was comparable to Ovx + FWA (10% w/w).

### 3.7 Modulation of osteoclast formation and activity in FWA (10% w/w) rats secures bone remodeling during the repair

Callus remodeling, which enables the conversion of cartilage to bone and remodeling of woven bone to lamellar bone, requires osteoclastic resorption. Osteoclast formation and activity were evaluated by histological examination of TRAP-stained fracture calluses 20-23 days post-fracture. This time frame coincides with the transition to the osteogenic phase of endochondral repair. FWA (10% w/w) on bone resorption was evaluated histologically by TRAP staining in bone tissue sections at 10X and 20X magnification. **Figure 7** demonstrates that PTH and FWA (10% w/w) treatments significantly improve fracture healing compared to the Ovx control group.

It was interesting to observe that FWA (10% w/w) treatment of fractured groups demonstrated its ability to modulate and reduce osteoclast numbers, as evidenced by less TRAP+ve stained cells ~40% in contrast to the ovariectomized group that presented with a large number of TRAP +ve osteoclast cells, while PTH showed no effect on osteoclasts as assessed by OC NO./BS (**Figure 7**).

RT-qPCR analyses confirmed the decrease in osteoclastogenesis by FWA (10% w/w). Expression of several osteoclast-specific genes, including TRAP (**Figure 7**), RANKL (**Figure 7**), and Cathepsin K (**Figure 7**) robustly down-regulated compared with the ovariectomized group.

This means FWA (10% w/w) supports the whole cycle of bone remodeling and the process of fracture repair, and therefore, it could be a much more effective therapeutic agent that can accelerate healing.

### 3.8 Impact of FWA (10% w/w) on the material properties of bone formed at the fracture site

**Figure 8:**
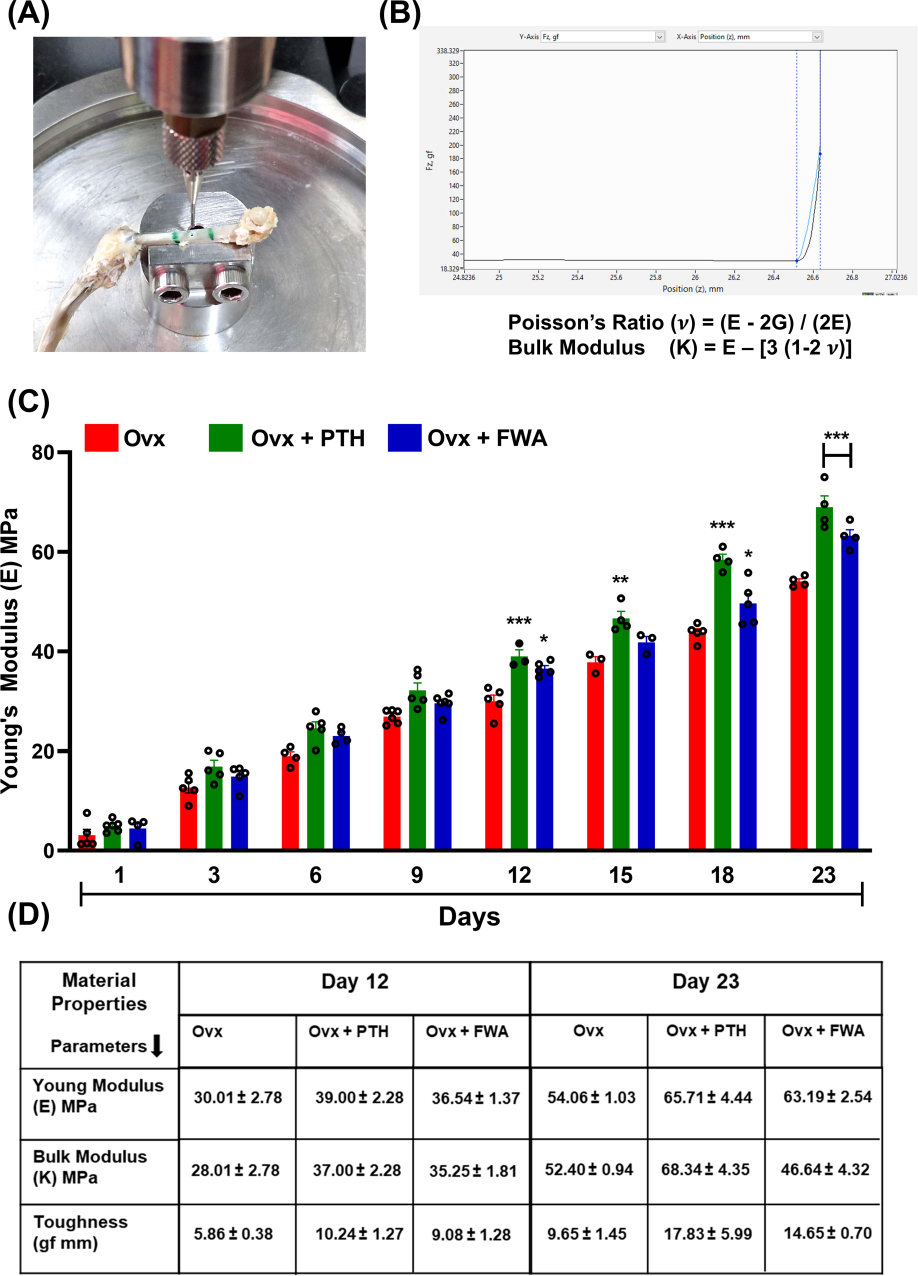
Biomechanical analysis of bone healing in ovariectomized (Ovx) rats treated with PTH or FWA. **(A)** Image showing the mechanical testing setup used to measure the biomechanical properties of healing tibiae, specifically Young’s modulus, using Biomomentum Mach 1 v500css. **(B)** Graph and equation illustrating the calculation of Poisson’s ratio (***v***) based on the relationship between Young’s modulus **(E)** and shear modulus **(G)**. This provides insights into the elastic deformation behaviour of bone under applied force. **(C)** Young’s modulus (E, MPa) of cortical bone from Day 1 to Day 23 post-fracture in Ovx models treated with Control (red), PTH (green), or FWA (blue). PTH and FWA treatment significantly improved Young’s modulus, showing the highest mechanical stiffness across all time points, particularly from Day 12 onwards. **(D)** Table summarizing key material properties (Young’s modulus, bulk modulus, and toughness) measured on Days 12 and 23. FWA-treated bones exhibited superior mechanical properties compared to the control, with higher Young’s modulus, bulk modulus, and toughness, especially on Day 23, indicating enhanced fracture healing. Data are presented as mean ± SEM, with statistical significance (***p < 0.001, **p 0.002, *p 0.05) compared to the Ovx control group.

To assess if the differential healing mechanisms affect the mechanical behaviour of the healing bone, the quality of bone being formed was assessed by nano-indentation testing to assess Young’s modulus (E) of the FWA (10% w/w) groups in comparison to the Ovx and PTH treated (positive control) groups. **Figure 8** shows the mechanical testing setup to assess the biomechanical properties of bone specimens, especially measuring bone material properties. The graph and equation highlight Poisson’s ratio calculation. Poisson’s ratio (**v**) is derived from the relationship between Young’s modulus (E) and shear modulus (G) using the formula.

**v** = (E−2G)/(2E). This provides insight into the elastic behaviour of the bone tissue under mechanical stress (**Figure 8**).


E=σ/ϵ

*where E is the Young’s modulus*

*σ is the ultimate stress (MPa)*

*ε is the strain*


Poisson's Ratio(v)=(E−2G)/(2E)

Bulk Modulus(K)=E–[3(1−2 v)]

The progression of Young’s modulus over time (Days 1-23) in three experimental groups under estrogen withdrawal conditions is represented in (**Figure 8**). FWA (10% w/w) treatment significantly improves the biomechanical properties of bone during healing in ovariectomized models, surpassing the effects of PTH. FWA (10% w/w)-treated bones exhibit superior stiffness, toughness, and overall mechanical integrity, indicating its potential as an effective therapeutic agent for enhancing bone healing and recovery, particularly under estrogen-deficient conditions (**Figure 8**).

### 3.9 Effect of FWA (10% w/w) on gene Expression at the fracture site

**Figure 9:**
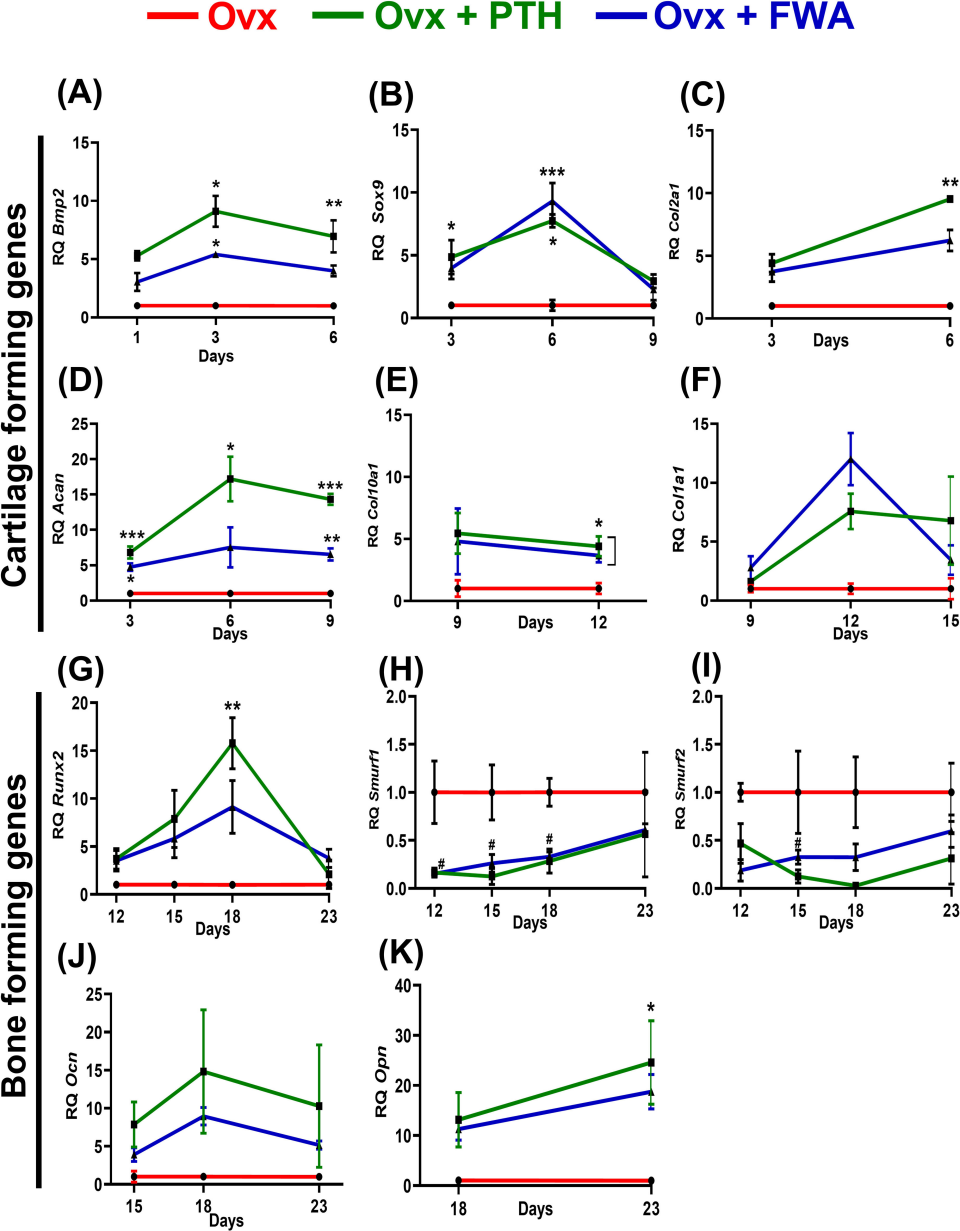
Analysis of gene expression levels in ovariectomized (Ovx) rats treated with Parathyroid Hormone (PTH) or Withania somnifera enriched extract (FWA). Quantitative analysis of gene expression levels over time for different treatment groups: Control (F, red), F + PTH (green), and F + FWA (blue) is shown with respect to different phases for BMP2 **(A)** Sox9 **(B)**, Col2a1 **(C)**, Acan **(D)**, Col1a1 **(E)**, Col10a1 **(F)**, Runx2 **(G)**, Smurf 1 **(H)**, Smurf 2 **(I)** Ocn **(J)**, Opn **(K)**. Statistical significance is denoted as follows: ***p< 0.001, **p 0.002, *p 0.033. Data are presented as mean ± SEM. ***p < 0.001, **p 0.002, *p 0.033, ^#^p 0.05. Data are presented as mean ± SEM. * indicate significant upregulation compared to the control group, while # denotes statistically significant downregulation relative to the control.

Compared to the Ovx fracture group, the mRNA level increased temporally, corresponding to the stages of inflammation, endochondral bone formation and remodeling phases. During the initial stages, periosteal cells exhibited a robust response to BMP2 (**Figure 9**), particularly noticeable on days 1, 3, and 6. Notably, on day 3, there was a significant increase in BMP2 gene expression, approximately (~9.0-fold) for PTH and about (~5.0-fold) for FWA (10% w/w), indicating a pronounced promotion of chondrogenesis followed by osteogenesis. As angiogenic cells began forming small blood vessels and fibrovascular tissue, upregulation of genes was observed in both PTH and FWA (10% w/w) at 125 mg.kg^-1^ by day 6. This was evidenced by notable increases in Sox9 (**Figure 9**) (~7.74-fold and ~9.31-fold respectively), Col2a1 (**Figure 9**) (~9.52-fold and ~6.22-fold respectively), and Aggrecan (**Figure 9**) (Acan) on day 9 (~17.20-fold and ~7.53-fold respectively), indicating progression towards endochondral bone formation. Subsequent stages involved the remodeling of bone tissue, marked by the expression of specific markers in both PTH and FWA (10% w/w) at 125 mg.kg^-1^. Notably, Col10a1 (**Figure 9**) exhibited increased expression on day 9 (~4.40-fold and ~3.67-fold, respectively), followed by Col1a1 (**Figure 9**) on day 12 (~7.57-fold and ~12.01-fold respectively). The expression of Runx2 (**Figure 9**) peaked on day 18 (~15.76-fold and ~9.11-fold, respectively), while that of PTH and FWA (10% w/w) decreased the expression of SMAD-specific E3 ubiquitin-protein ligases which degrades Runx2, at day 18. We observed that Smurf 1 was decreased by ~0.2835-fold and ~0.3296-fold respectively (**Figure 9**) and Smurf 2 by ~0.026-fold and ~0.3240-fold respectively (**Figure 9**), coinciding with mineralized marker expression such as Ocn (**Figure 9**) on day 18 (~14.81-fold and ~8.95-fold respectively) and Opn (**Figure 9**) on day 23 (~24.59-fold and ~18.73-fold respectively). These findings underscore a coordinated cascade of events leading to bone formation and maturation.

## 4 Discussion

This study examines the effectiveness of FWA (10% w/w) at different dosages and strengths on fracture healing, including the potential of its acceleration in callus formation and mineralization at the fracture site in healthy and estrogen-cut-down (Menopausal) rats.

Our findings show that a dose of 125 mg.kg^-1^ of FWA (10% w/w) enrichment consistently improved key indices of fracture healing, including total callus volume, mineralized tissue volume, and bone volume, as compared to other doses and PTH treatment.

**Figure 10:**
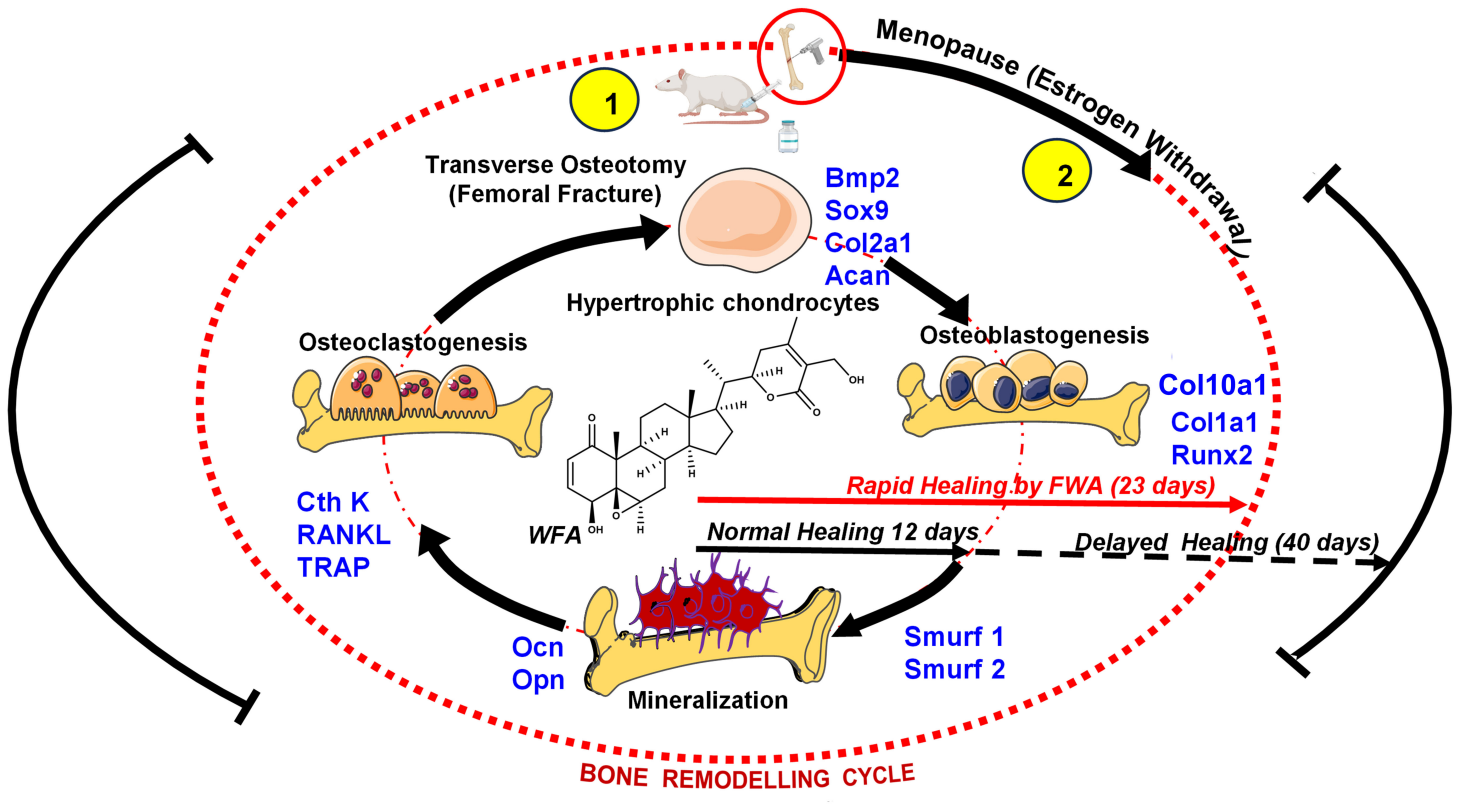
Schematic representation to show how FWA administration in rodents modulates the bone remodeling cycle at the molecular level to accelerate bone formation at the time of menopause (estrogen deficiency) and in fractures (transverse osteotomy).

At the fracture site, administration of FWA (10% w/w) led to a notable decrease in the activity of osteoclast marker genes, indicating reduced bone resorption. Simultaneously, there was an increase in anabolic responses, as evident by including heightened expression of osteogenic markers (25) and by downregulating the expression of E3 ubiquitin ligase genes Smurf 1 and Smurf 2 which are responsible for degradation of Runx2, a key transcription factor for bone formation phase. By keeping Smurf 1 and 2 levels significantly low, FWA (10% w/w) may have provided early bone-forming effects in different physiological models. These molecular changes facilitated endochondral ossification and bone remodeling, resulting in a remarkable increase in bone volume/tissue volume (BV/TCV %) by day 12, significantly faster than the normal healing process that takes 21-35 days for complete fracture healing in the rodents (**Figure 10**) (24).

The pharmacokinetic profiling of FWA (10% w/w) at 125 mg.kg^-1^ confirmed an optimized formulation enriched with Withaferin A (WFA), as evidenced by its high plasma concentration (Cmax) and area under the curve (AUC). Withanolide profiling revealed rapid absorption and a prolonged half-life for WFA, suggesting it may drive FWA’s (10% w/w) efficacy in fracture repair. The Tmax and extended plasma half-life of WFA indicate favourable pharmacokinetics for fracture healing applications. These properties, along with FWA’s (10% w/w) favourable log P and total polar surface area, suggest an optimal balance of lipophilicity and permeability conducive to its therapeutic activity in rapid fracture models.

The effectiveness of FWA (10% w/w) was compared to Parathyroid Hormone (PTH), a well-known stimulator of bone healing (26). Animal experiments show that PTH can improve normal fracture healing (27, 28). While PTH increased Bone Volume to completion, FWA (10% w/w) was also able to achieve almost the same value (~95%). This nearly equivalent bone-regeneration by FWA (10% w/w) suggests that it could be a potent alternative for PTH, with reduced side effects. PTH, which primarily aided bone healing through bone modelling. Studies show that PTH treatment in mice delays the complete replacement of cartilage anlagen by endochondral bone during skeletal repair due to the inhibitory effect of PTH on chondrocyte maturation. However, it improves biomechanical properties by enhancing osteoblastogenesis and new bone formation (13). In contrast, FWA (10% w/w) effectively reduced osteoclast activity and promoted bone remodelling, indicating a more balanced and comprehensive approach to bone regeneration. Bone stiffness has also been shown to be significantly influenced by the degree of bone mineralization; several studies have described a direct relationship between Young’s modulus, a material index of stiffness, and bone mineral density (29, 30).

In a menopausal model, which mimics delayed healing due to estrogen deficiency, FWA (10% w/w) showed significant callus mineralization over the time period, achieving significant increases in BV/TCV by day 23 and underscoring its potential to support fracture repair under osteoporotic conditions. Histological analysis confirmed greater mineralized callus volume and reduced cartilage proportions in the FWA (10% w/w) and PTH groups compared to controls, suggesting that FWA (10% w/w) facilitates endochondral ossification. FWA (10% w/w) restored the callus area and promoted bone healing with better mechanical strength (Young’s Modulus). These positive results were accomplished within 23 days, thus shortening by approximately 30% from the standard healing time to 35-50 days (31).

This study highlights the promising potential of wound-healing enriched *Withania somnifera* extract (FWA, 10%w/w) in accelerating fracture healing and improving bone regeneration in typical and estrogen-deficient (menopausal) conditions. FWA (10% w/w) has been found to show excellent improvements in callus formation and mineralization that are very effective in stimulating osteoblast activity and thereby balancing osteoclast function for effective healing. FWA (10% w/w) is a less expensive and accessible alternative with broad therapeutic potential in addressing the limitations of conventional therapies. These findings, therefore, provide a robust foundation to advance FWA (10% w/w) into clinical applications and pave the way for its use as a novel agent in bone health management.

This study highlights the efficacy of FWA (10% w/w) in promoting fracture healing; however, several areas merit further exploration to enhance its practical application. Based on the pharmacokinetics observed in this research, investigating alternative drug delivery methods with FWA (10% w/w) —such as nano formulations, or combination therapies with existing therapeutic agents—could significantly improve bioavailability and its potential.

However, it is essential for future studies with FWA (10% w/w) beyond current preclinical models that includes large-animal models for better simulate human bone physiology. Conducting clinical trials to evaluate the efficacy of FWA (10% w/w) in patients with osteoporosis or those experiencing impaired fracture healing will be vital for translating these findings into clinical practice. Addressing these aspects will further validate FWA’s (10% w/w) potential as a cost-effective, safe, and effective therapeutic option for bone repair and fracture healing.

## 5 Conclusion

The findings summarize FWA’s (10% w/w) potential to support the biological process of bone remodeling by modulating osteoclast activity, promoting osteoblast differentiation, and enhancing the mechanical integrity of healing bone. Such characteristics place FWA (10% w/w) in a lead position as a therapeutic agent to hasten fracture healing, especially in delayed bone repair.

Besides overcoming the excessive expense of pure Withaferin A, FWA (10% w/w) has several other benefits. Its enriched composition increases bioavailability and prolonged plasma concentration, corresponding to more efficient therapeutics’ effects. In contrast to common anabolic drugs like Parathyroid Hormone (PTH), Which mainly include bone growth but have little impact on osteoclast regulation, FWA (10% w/w) reveals a greater therapeutic potential in enhancing osteogenesis while suppressing uncontrolled osteoclast activity, thus ensuring harmonious remodeling of bones. This is particularly evident in osteoporotic fractures, where enhanced bone resorption can result in delayed healing or non-union.

Additionally, the effectiveness of FWA (10% w/w) in inhibiting systemic inflammation offers another benefit, as chronic inflammation plays a significant role in impaired bone repair in postmenopausal osteoporosis and other skeletal diseases. Its capacity to advance the process from the fibrovascular stage towards bone remodeling sets it apart as a treatment with the potential to reduce recovery time.

Further, FWA’s (10% w/w) beneficial pharmacokinetics, such as slow plasma half-life and ideal absorption rate, underpin its potential to be given orally- providing a minimally invasive alternative to injectable anabolic drugs currently employed in fracture repair. Such properties and their efficacy in estrogen-deficient states indicate that FWA (10% w/w) may be versatile therapeutic agent for generalised clinical use in managing bone metabolic disorders.

## Data Availability

The raw data supporting the conclusions of this article will be made available by the authors, without undue reservation.
